# High Prevalence of High-Risk HPV Among People with and Without HIV: Insights into Risk Factors for Tailored Screening Approaches

**DOI:** 10.3390/microorganisms12122571

**Published:** 2024-12-13

**Authors:** Elena Bruzzesi, Federica Gandini, Sara Diotallevi, Riccardo Lolatto, Massimo Cernuschi, Caterina Candela, Angelo Roberto Raccagni, Flavia Passini, Andrea Marco Tamburini, Roberto Burioni, Antonella Castagna, Silvia Nozza

**Affiliations:** 1 Infectious Diseases Unit, Vita-Salute San Raffaele University, 20132 Milan, Italycandela.caterina@hsr.it (C.C.); raccagni.angelo@hsr.it (A.R.R.); passini.flavia@hsr.it (F.P.); burioni.roberto@hsr.it (R.B.); castagna.antonella@hsr.it (A.C.); nozza.silvia@hsr.it (S.N.); 2 Infectious Diseases Unit, IRCCS San Raffaele Scientific Institute, 20132 Milan, Italy; diotallevi.sara@hsr.it (S.D.); lolatto.riccardo@hsr.it (R.L.); cernuschi.massimo@hsr.it (M.C.); 3 Gastrointestinal Surgery Unit, IRCCS San Raffaele Scientific Institute, 20132 Milan, Italy; tamburini.andreamarco@hsr.it; 4 Laboratory of Microbiology and Virology, IRCCS San Raffaele Scientific Institute, 20132 Milan, Italy

**Keywords:** human papillomavirus, 9vHPV, HPV vaccine, HIV-1, men who have sex with men, nonavalent vaccine

## Abstract

Men who have sex with men (MSM) and people with HIV are at increased risk of anal HPV infection and cancer. This study aimed to assess the prevalence of anal HPV among MSM with HIV (MWH) and without HIV (MWoH), as well as among MSM under and over 35 years. Factors associated with infection from high-risk (HR) HPV were investigated. This retrospective cohort analysis included MSM receiving care at IRCCS San Raffaele, Milan, Italy, with at least one HPV test collected from 2014 to 2023. Among 1577 MSM, 1427 (90%) were MWH. At first screening, 87.6% were had HR-HPV and MWoH were significantly younger, as compared to MWH. Cytological abnormalities were more frequent among younger MSM, while high-grade lesions were more frequent among those over 35 years of age. In multivariate regressions, the risk of ≥1 HR-HPV genotype at first and last screening was associated with younger age (adjusted odds ratio, aOR (95% confidence interval): 0.33 (0.18, 0.59); 0.34 (0.18, 0.58), *p* < 0.001) and concomitant STI (aOR 2.74 (1.59, 5.08), *p* < 0.001; 1.68 (1.09, 2.67), *p*: 0.023). A discrepancy between the recommend and actual age for screening was found. As a younger age and concomitant STIs were associated with infection by ≥1 HR-HPV, we promote a more tailored screening approach for both MWH and MWoH.

## 1. Introduction

Human papillomavirus (HPV) is a global public health concern and one of the most common causes of sexually transmitted disease in both men and women. HPV infects epithelial cells of the skin and mucosal tissues, including the cervix, vagina, anus and throat, potentially leading to cancer.

It is also well established that more than 80% of sexually active individuals will be exposed to and acquire HPV at some point in their lifetime [1]. In particular, men who have sex with men (MSM) and people with HIV are at elevated risk for acquiring HPV at the anal site. According to a pooled analysis from 64 studies, HR-HPV prevalence was 6.9% among men who have sex with women without HIV and 26.9% among those with HIV, rising to 41.2% among MSM without HIV and 74.3% among those with HIV [2,3]. Indeed, HIV induces immune suppression by targeting and depleting CD4+ T cells, which are crucial for orchestrating immune responses, thereby impairing the body’s ability to clear HPV infections [4]. However, the exact mechanism by which HIV contributes to HPV-induced carcinogenesis remains to be fully elucidated. On the other hand, HPV infection appears to increase the susceptibility to HIV acquisition by disrupting the mucosal barrier and promoting immune activation [5]. Other key risk factors for HPV infection include number of sexual partners, age at sexual debut, use of oral contraceptives, co-infections with other sexually transmitted viruses and immunodepression (e.g., due to HIV, defects in T cell-mediated immunity, use of immune-suppressive agents and organ transplantation). The state of immunodepression, along with smoking, age and the presence of precancerous lesions, contributes to cancer progression [6,7,8]. Numerous studies have demonstrated that MSM living with HIV are at the highest risk for developing anal cancer; a meta-analysis estimates the incidence of anal cancer at 85 per 100,000 person-years, with age-specific rates ranging from 16.8 per 100,000 for those <30 years to 107.5 per 100,000 for those ≥60 years. Among MSM living with HIV, the incidence is 32 per 100,000 person-years [9].

Recent guidelines from the International Anal Neoplasia Society (IANS) recommend that anal cancer screening in MSM with HIV should start at age 35, while screening for MSM without HIV should start at age 45, without considering other risk factors [10]. On the other hand, the National Institutes of Health (NIH) HIV Clinical Guidelines endorse the IANS recommendations regarding the age to begin screening, but also suggest considering additional risk factors to prioritize individuals for high-resolution anoscopy [11]. Notably, CD4 nadir has been recently shown to be associated with cancer progression among people with HIV [12,13]. Moreover, the ANCHOR trial showed that detecting and treating precancerous anal lesions reduce the progression to anal cancer [14]. Thus, it is essential to identify individuals at increased risk of anal infection and cancer progression, who would benefit the most from screening.

In this paper, we will focus on analyzing the prevalence of HPV serotypes at the anal site at first screening in MSM. We will compare risk factors for anal infection from high-risk (HR) HPV genotypes in MSM with HIV (MWH) and without HIV (MWoH). Then, we will assess the prevalence of HPV genotypes and cytological abnormalities at the most recent test. Risk factors will be compared between MSM under 35 years of age and those over 35, with the latter aligning with the recommended age for initiating screening.

After providing an overview of the cohort, our goal is to identify factors associated with the risk of anal infection caused by high-risk HPV (HR-HPV) genotypes. This will help us better characterize the population that could benefit from more targeted screening strategies.

## 2. Materials and Methods

### 2.1. Study Population and Characteristics

This is a retrospective observational study conducted on a cohort of MSM living with or without HIV, receiving care at the Infectious Diseases Unit of the San Raffaele Hospital, Milan, Italy. We included men who self-reported as MSM who underwent screening for anal carcinoma in the time period between January 2015 and December 2023 (data lock).

To assess prevalence and describe the characteristics of MWH and MWoH at first screening, individuals with prior HPV tests were excluded. To compare HPV prevalence and clinical characteristics among MSM under and over 35 years, we considered the last available HPV test. In particular, to characterize the population independent of HPV vaccination status and to mitigate potential bias arising from recall error, we only included individuals who had an HPV test conducted prior to receiving the HPV vaccination.

Laboratory parameters determined at or within 180 days prior to HPV testing were CD4 T cell count and percentage, CD8 T cell count and percentage, CD4/CD8 ratio and viremia and concomitant STI. History and concomitant STIs include syphilis, hepatitis C and B infections, Neisseria gonorrhoeae, Chlamydia trachomatis and mpox. Syphilis and hepatitis C and B infections were documented by serology, Neisseria gonorrhoeae by culture tests and nucleic acid amplification techniques and Chlamydia trachomatis, Herpes viruses and mpox by nucleic acid amplification techniques.

Anonymized data were collected in the electronic health records Centro San Luigi (CSL) Cohort database (CSL-Cohort). The CSL Cohort was approved by the Ethics Committee of the IRCCS San Raffaele Scientific Institute, Milan, Italy (date of approval: 4 December 2017, protocol n. 34). Recorded data were managed according to Good Clinical Practice and include demographics, clinical history (i.e., years of HIV infection, naïve status, years of antiretroviral therapy, type of highly active antiretroviral therapy regimen, and years of viral suppression defined as viremia maintained below 50 copies/mL), and virologic and laboratory data.

### 2.2. Standard Anoscopy and HPV Genotyping

HPV screening involves a digital rectal examination, followed by standard anoscopy; at screening, co-testing with an HPV test and cytology was performed.

During anoscopy, a cytobrush was introduced into the anal canal and rotated with circular motions to collect cells for cytology examination, then smear-swabbed on a slide and fixed with a cytological fixative spray. Cytology samples were analyzed using Papanicolaou staining, following standard procedure.

Subsequently, a cotton Dacron swab was inserted into the anal canal, gently rotated in a circular motion about 2–3 cm from the anal verge, then placed in a thin-layer liquid medium for HPV testing. DNA was extracted from 200 µL from each specimen using the semi-automated NIMBUS extraction platform (Seegene, Seoul, Republic of Korea). Genotyping was performed by Anyplex^TM^ (Seegene, Seoul, Republic of Korea), a method which relies on multiplex real-time polymerase chain reaction, simultaneously detecting 28 HPV genotypes (LR-HPV genotypes: 6, 11, 40, 42, 43, 44, 54, 61, 70; HR-HPV genotypes: 16, 18, 26, 31, 33, 35, 39, 45, 51, 52, 53, 56, 58, 59, 66, 68, 69, 73, 82). Sequence amplification was performed by a CFX96TM RT-PCR system (Seegene, Seoul, Republic of Korea).

According to the up-to-date terminology, the examined cytological specimens were classified using a two-grade nomenclature into low-grade and high-grade squamous intraepithelial lesions (LSILs and HSILs) or using a three-grade nomenclature into anal intraepithelial neoplasia (AIN) grade 1, 2 or 3. In the case of AIN1, the lesion was classified as LHSIL; in the case of AIN2 or 3, it was classified as an HSIL. Other lesions, such as atypical squamous cells of undetermined significance (ASCUSs), infection and condylomata, were also reported [15].

### 2.3. Statistical Analyses

Qualitative variables were reported as absolute values and frequency (%), while quantitative variables as median and interquartile range (IQR). Quantitative variables were compared using the Mann–Whitney test. Differences between proportions were tested by the chi-square (χ^2^) or Fisher’s exact test. Multivariable logistics regressions were used to estimate risk factors associated with the presence of high-risk HPV. Variables considered clinically relevant were included in the model. Adjusted odds ratios were reported with the corresponding 95% confidence interval.

All statistical tests were two-sided at the 5% level and were carried out using R Statistical Software, version 4.2.3 (R Foundation for Statistical Computing, Vienna, Austria).

## 3. Results

### 3.1. Anal Cancer Screening: Characteristics at First HPV Test

A total of 1577 MSM underwent their first HPV test between January 2015 and December 2023 and were included in the analyses: 1427 (90.5%) were MWH and 150 (9.5%) were MWoH. The median age at which this first test was conducted was 43.8 years.

**Figure 1:**
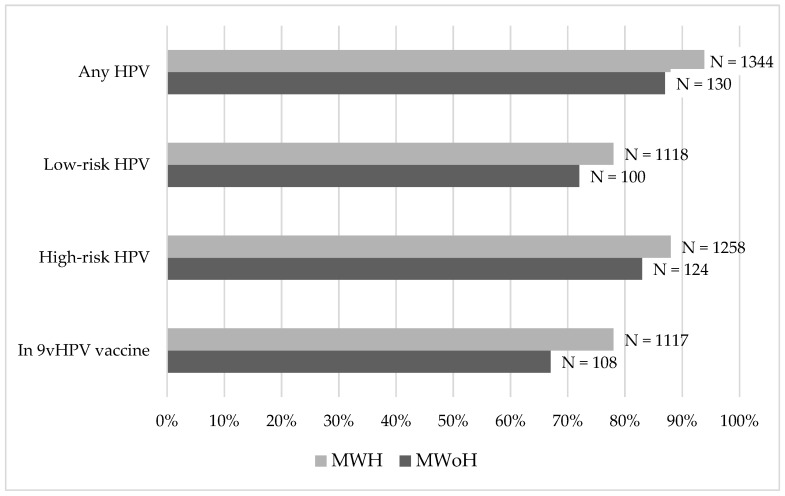
Percentage and number (N) of MSM with HIV (MWH) and MSM without HIV (MWoH) who tested positive for any of the following: HPV, LR-HPV, HR-HPV and the serotypes covered by the 9-valent vaccine at the first HPV test.

At the first HPV test, 1474 (93.5%) MSM had at least one HPV serotype detected: 130 (86.7%) MWoH and 1344 (94.2%) MWH (Figure 1). Prevalence for any genotype and genotypes included in the nonavalent vaccine was higher in MWH, as compared to MWoH (*p*: 0.001 and *p*: 0.002, respectively). Prevalence of HPV-16 was similar between the two groups (24.0% vs. 27.6%, *p*: 0.396). The prevalence of each HPV serotype tested is reported in Figure S1.

Among the 1427 MWH, 1409 (98.7%) were on antiretroviral therapy (ART) at testing. Immunovirological parameters were optimal, with 89.9% of individuals exhibiting undetectable viremia and a median CD4 T cell count of 737 cells/µL (IQR 554–940). The median CD4/CD8 ratio was 0.80 (0.56–1.10). Additionally, 154 individuals (10.8%) had a history of AIDS, with a median CD4 T cell nadir of 355 cells/µL. Specifically, 80.3% (1120) of MWH had a nadir CD4+ count greater than 200 cells/µL, while 19.7% (274) had a count of 200 cells/µL or less. HPV testing was conducted a median of 8.5 years after HIV diagnosis. Detailed characteristics for people living with HIV (PWH) are reported in Table S1.

At screening, MWH were significantly older than MWoH, while smoking and vaccination status was not different in the two populations (Table 1).

**Figure 2:**
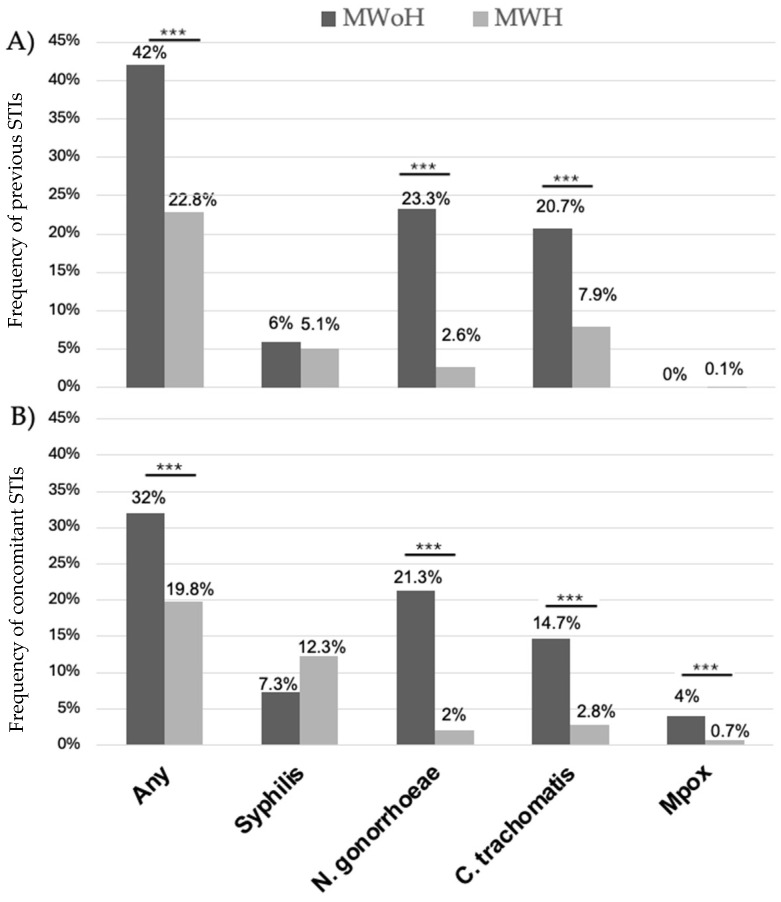
History (panel (**A**)) or concomitant (panel (**B**)) STIs were more frequent among MWoH than MWH at HPV screening. *** indicates *p* < 0.001.

In contrast, concomitant and previous STIs were more frequent among MWoH than MWH. This difference was significant in cases with a history or concomitance of Chlamydia and gonorrhea and in cases with a history of mpox (Figure 2).

### 3.2. Prevalence of HPV Genotypes at Anal Site and Cytological Abnormalities Before Vaccination in People Over and Under 35 Years of Age

In accordance with IANS guidelines for anal carcinoma screening, this analysis aims to compare HPV test results between MSM under the age of 35 and those aged 35 and older. The study included 563 MSM who underwent at least one HPV test before receiving HPV vaccination: 506 (89.9%) were MWH, while 57 (10.1%) were MWoH. The median age at the time of testing was 37.4 years. On average, HPV tests were conducted 10.1 months prior to the administration of the first vaccine dose, with a mean interval of 9.2 months for the under-35 cohort and 10.9 months for those over 35.

**Figure 3:**
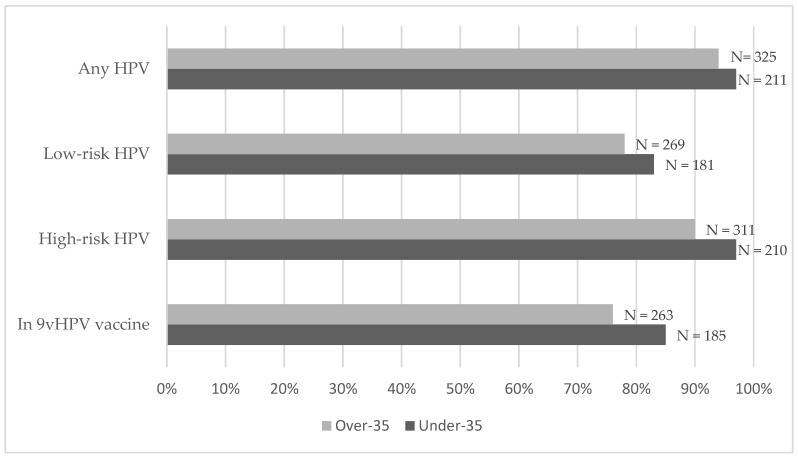
Percentage and number (N) of MSM who tested positive for any of HPV, LR-HPV, HR-HPV and the serotypes covered by the 9vHPV vaccine at their last HPV test before immunization.

Overall, 536 patients (95.2%) tested positive for at least one HPV strain in their last test. Specifically, 97.2% (211 individuals) of the under-35 group tested positive, compared to 93.9% (325 individuals) of the over-35 group (Figure 3). The prevalence of genotypes included in the 9vHPV vaccine and that of HR-HPV were significantly higher in the under-35 population (*p* = 0.004 and *p* = 0.011, respectively). On the other hand, the prevalence of HPV-16 was similar between the two groups (33.6% in the under-35 group versus 25.7% in the over-35 group, *p* = 0.054). Detailed prevalence data for each HPV serotype are reported in Figure S2.

Furthermore, cytological testing was performed in 514/563 (91.3%) individuals. Abnormalities were more frequent among MSM younger than 35; however, the frequency of high-grade lesions was higher in the over-35 group.

As far as HIV infection is concerned as a risk factor, 505/506 individuals were on ART. Immunovirological parameters were optimal at testing, with no substantial differences between the two groups; 92.2% had undetectable viremia, the median CD4 T cell count was 748 (588–939) and the median CD4/CD8 ratio was 0.84 (0.60–1.10). Additionally, AIDS was diagnosed in only 25 cases (5%), including 10 cases (5.2%) in the under-35 group and 15 cases in the over-35 group. Median CD4 T cell nadir was 405 cells/µL, specifically 441 cells/µL for those under 35 and 387 cells/µL for those over 35 (Table 2).

Previous STIs were more frequent among MWoH than MWH (Figure 2).

**Figure 4:**
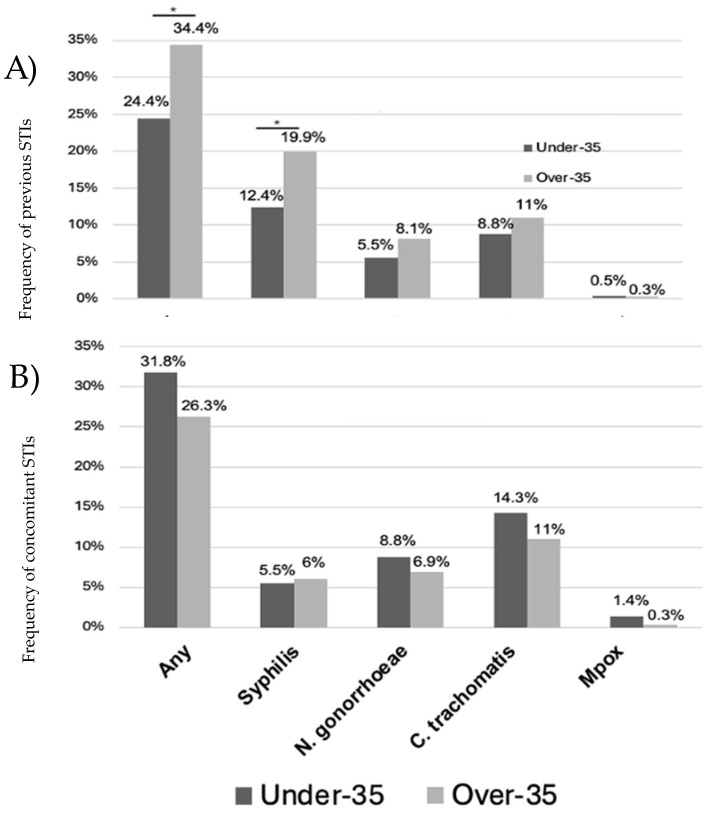
History (panel (**A**)) or concomitant (panel (**B**)) STIs were more frequent among MWoH than MWH at last HPV test before immunization. * Indicates significance at *p* < 0.05.

A total of 172 individuals (30.6%) had a history of at least one STI at the time of the HPV test, namely 24.4% of people under 35 (53 individuals) and 34.4% of people over 35 (119 individuals). In addition, 160 (28.4%) had a concomitant history of at least one other STI, namely 31.8% (69) of under-35 individuals and 26.3% (91) of over-35 individuals (details for syphilis, gonorrhea, Chlamydia and mpox in Figure 4).

### 3.3. Risk Factors Associated with HR-HPV

Regarding multivariate regression, after adjustment for each STI (including HCV, HBV, gonorrhea, Chlamydia, syphilis and mpox), the risk of ≥1 HPV-HR genotype was associated with younger age and concomitant STI, both at screening and last HPV test before immunization (Table 3).

## 4. Discussion

A significant prevalence of at least one HPV strain was observed, affecting 93.5% of the population, with 94.2% among MWH and 86.7% among MWoH. Notably, we found that 87.6% of participants tested positive for at least one HR-HPV, with rates of 88.2% for MWH and 82.7% for MWoH. These findings align with previous studies: in particular, a meta-analysis reported a prevalence of 92.6% for any HPV genotype at the anal site among MWH, with 73.5% for HR-HPV genotypes [16]. Similarly, HPV prevalence in the IPERGAY cohort, which includes MWoH on pre-exposure prophylaxis (PrEP), was reported as 92% for any HPV and 84% for HR serotypes [17]. Additionally, high prevalence rates of HPV at the anal site have been documented in Italy [18,19]. Our results further corroborate findings from a study involving MSM and transgender individuals in Southeast Asia, which reported a higher prevalence of any HPV among MWH (89.8%), compared to MWoH (65.3%) [20].

We also found that the MSM included in our cohort were offered their first anal HPV test at a median age of 43.5 years. On the other hand, MWoH received screening at a younger age with respect to MWH (39.7 vs. 44.5 years, respectively). This finding is in contrast with the recently published IANS and NIH guidelines, which recommend that anal carcinoma screening should begin at age 35 for MWH, due to the augmented risk in this population [10,11,21]. However, in other cohorts including MSM, the median age at which HPV testing is performed is 43 years for MWH and 35 years for MWoH [17,22], demonstrating a discrepancy between clinical practice and the recently published guidelines.

Among immunized individuals, HPV was positive prior to the vaccine in more than 95% of cases. Moreover, cytological abnormalities were found in 16.5% of cases, with a higher percentage in MSM under 35 (18.2%). In turn, high-grade lesions were more frequent in the over-35 population. According to a meta-analysis, anal HSIL was more frequent among MWH (22%) than among women (13%) and MWoH (12%). However, the prevalence of HSIL within the MSM population showed considerable variability, ranging from 3% to 50%, attributable to factors such as temporal factors, variability in clinical practice and HRA expertise [23]. Taking into consideration the data on the prevalence of HPV and the associated lesions in the under-35 population, we reinforce the indication to an earlier vaccine prophylaxis to limit the spread of HPV.

Moreover, the immune system status is a determinant of susceptibility to HPV infections and of cancer progression. Individuals who have undergone organ or bone marrow transplants, those with inherited immune deficiencies or autoimmune disorders, and people living with HIV are at higher risk of anal cancer [24]. Indeed, we have shown a higher prevalence of any HPV genotype among MWH than among MWoH (94.2% vs. 86.7%). However, the prevalence of HPV-16 was similar. In detail, concerning the immune–virological characteristics of MWH, we found that CD4 nadir and count were not risk factors for having HR-HPV genotypes at multivariate regression, which is consistent with other studies but in contrast to some others [25,26]. Indeed, the correlation between CD4 nadir and disease progression to cancer has recently been demonstrated [10,11].

Similarly, smoking was also found to not be significantly associated with the risk of having at least one HR-HPV.

Another key finding is that MWoH, despite being younger, were more likely to have a history of STIs (32% vs. 19.8% for MWH) or concomitant (42% vs. 22.8% for MWH) STIs at the time of HPV testing. Moreover, in the cohort of vaccinated patients, individuals over 35 were more likely to have a history of STIs, while younger individuals were more frequently found to have a concomitant STI at the time of testing.

These data could relate to greater sexual activity, more partners and an earlier sexual debut among the younger population and in MWoH. Interestingly, data from a Brazilian cohort of young men showed that HPV/STI coinfection was more prevalent than HPV infection alone: the most frequently associated STI was gonorrhea, in accordance with our results [27]. In fact, gonorrhea leads to an extracellular inflammatory infection, which is linked to elevated levels of inflammatory cytokines and tumor necrosis factor [28]. This environment may promote the entry and persistence of HPV [29]. Furthermore, gonorrhea has been associated with HR-HPV infection and with the progression to high-grade squamous intraepithelial lesions, especially in the presence of HIV and high-risk HPV. This suggests a potential interaction between these infections that may accelerate disease progression [30,31].

Moreover, similar to cervical screening, anal HPV testing demonstrates higher sensitivity compared to anal cytology alone. However, given the high HPV prevalence in the study population, reliance on HPV testing alone could reduce specificity. In fact, in our cohort of vaccinated individuals, co-testing was performed in 91.3% of cases. Consequently, we advocate for the NIH guidelines, which recommend the concurrent use of both tests [11].

Finally, only 27.4% of MSM were vaccinated for HPV at screening; given that the median year at testing was 2018, vaccination coverage was suboptimal. However, data from other cohorts report even lower rates; namely, 17% and 15% among MSM vaccinated before the age of 26 in the United States and France [32,33]. Although data on HPV vaccination coverage remain limited, existing information is concerning and underscores the need to sustain vaccination programs targeting MSM, which have been hindered by vaccine shortages and the COVID-19 pandemic. Moreover, it is crucial to allocate resources to enhance awareness among MSM about the risk of HPV-related diseases and the importance of vaccination.

This study has several strengths. Notably, it assesses a large cohort of MSM receiving care at a tertiary center, with data that span for over 10 years. To our knowledge, this is the first study to assess HPV infection among MSM, examining their characteristics based on age. However, we acknowledge some limitations. First, the study population consisted of MWoH who voluntarily presented at IRCCS San Raffaele either as PrEP users or seeking care for other STIs. As such, they may not be representative of the entire MWoH population, which introduces potential selection bias. Additionally, other limitations include the retrospective design and the lack of information on sexual practices and number of partners. Collecting data on sexual behavior is challenging due the limitations of measurement techniques: self-report methods are vulnerable to biases, such as recall bias. Additionally, the use of categorical classifications may fail to capture the full spectrum of sexual behaviors.

## 5. Conclusions

In this study, we found that young MSM, particularly those with concurrent STIs, are at an elevated risk for HR-HPV infection. Furthermore, we observed a high prevalence of HR-HPV and HPV-associated lesions prior to immunization. Consequently, we reinforce the recommendations established by the NIH and IANS [10,11] to provide screening for MSM, regardless of HIV status, and to continue offering early prophylactic measures.

However, in light of the findings from the ANCHOR trial, we propose that a more personalized screening approach should be considered, where feasible. This approach would involve evaluating additional risk factors for HPV acquisition, beyond age, focusing on STIs, especially among MWoH. Therefore, further studies are warranted to enhance the identification of target populations that could benefit from a more individualized screening strategy.

## Figures and Tables

**Table 1:** Characteristics of MSM without HIV compared with MWH at HPV testing.

Variable	TOT N = 1577	MWoH N = 150	MW N = 1427	*p* Value
Age	43.8 [35.2; 52.1]	39.7 [33.3; 47.8]	44.5 [35.5; 52.4]	<0.001
9vHPV vaccination status	432 (27.4%)	39 (26.0%)	393 (27.5%)	0.759
History of STI	330 (20.9%)	48 (32.0%)	282 (19.8%)	0.001
At least one concomitant STI	388 (24.6%)	63 (42.0%)	325 (22.8%)	<0.001
History of HCV	132 (8.79%)	2 (1.83%)	130 (9.34%)	0.013
History of HBV	71 (5.51%)	0 (0.00%)	71 (5.71%)	0.171
Smoking				0.058
Yes	512 (43.3%)	9 (26.5%)	503 (43.8%)	
Never	521 (44.1%)	22 (64.7%)	499 (43.5%)	
Former smoker	149 (12.6%)	3 (8.82%)	146 (12.7%)	

**Table 2:** Characteristics of MSM without HIV compared with MWH at last HPV test before HPV vaccine.

Variable	TOT N = 563	MSM < 35 N = 217	MSM ≥ 35 N = 346	*p* Value
Age	37.4 [31.8; 42.2]	30.2 [27.9; 32.7]	41.1 [38.2; 44.6]	<0.001
Years from HIV diagnosis	6.77 [2.59; 11.4]	3.26 [1.20; 6.95]	9.17 [4.94; 13.2]	<0.001
Years of ART	5.21 [2.03; 8.58]	2.34 [0.92; 5.81]	6.87 [3.71; 10.2]	<0.001
History of AIDS	25 (4.94%)	10 (5.21%)	15 (4.78%)	0.995
HIV-RNA (copies/mL)	0.90 [0.90; 38.8]	0.90 [0.90; 39.0]	0.90 [0.90; 23.0]	0.937
Undetectable HIV-RNA	426 (92.2%)	156 (90.2%)	270 (93.4%)	0.279
Years of viral suppression	2.12 [0.60; 6.10]	1.17 [0.34; 2.78]	3.80 [0.83; 7.92]	<0.001
Nadir CD4+ (cells/microL)	405 [269; 573]	441 [319; 626]	387 [251; 549]	0.001
Nadir CD4+ >200 cells/microL	426 (85.2%)	168 (89.4%)	258 (82.7%)	0.057
CD4+ (cells/microL)	748 [588; 939]	743 [566; 894]	758 [602; 963]	0.373
CD4%	32.0 [26.4; 37.7]	32.0 [25.8; 37.4]	31.8 [27.2; 38.3]	0.453
CD8+ (cells/microL)	909 [686; 1171]	893 [684; 1190]	918 [688; 1156]	0.718
CD8%	37.8 [32.2; 45.9]	37.7 [32.8; 46.5]	38.0 [31.9; 45.8]	0.910
CD4/CD8 ratio	0.84 [0.60; 1.10]	0.82 [0.58; 1.09]	0.84 [0.61; 1.11]	0.644
Type of ART regimen				0.052
NNRTI-based	141 (28.0%)	40 (21.1%)	101 (32.3%)	
PI-based	33 (6.56%)	14 (7.37%)	19 (6.07%)	
INSTI-based	297 (59.0%	124 (65.3%)	173 (55.3%)	
NRTI only	1 (0.20%)	0 (0.0%)	1 (0.32%)	
Other	28 (5.57%)	11 (5.79%)	17 (5.43%)	
Off-therapy	2 (0.40%)	0 (0.0%)	2 (0.64%)	
Cytologic abnormalities				0.033
Negative	429 (83.5%)	157 (81.8%)	272 (84.5%)	
Atypical squamous cells of undetermined significance	12 (2.33%)	9 (4.69%)	3 (0.93%)	
Low-grade squamous intraepithelial lesion	48 (9.34%)	19 (9.90%)	29 (9.01%)	
High-grade squamous intraepithelial lesion	10 (1.78%)	1 (0.52%)	9 (2.60%)	
Condylomata/infection	15 (2.66%)	6 (3.12%)	9 (2.60%)	
History of STI	172 (30.6%)	53 (24.4%)	119 (34.4%)	0.016
At least one concomitant STI	160 (28.4%)	69 (31.8%)	91 (26.3%)	0.190
History of HCV	45 (8.33%)	5 (2.4%)	40 (12.0%)	<0.001
History of HBV	16 (3.57%)	4 (2.33%)	12 (4.35%)	0.390
Smoking				0.093
Yes	182 (43.5%)	69 (49.3%)	113 (40.6%)	
Never	197 (47.1%)	63 (48%)	134 (48.2%)	
Former smoker	39 (9.33%)	8 (5.71%)	31 (11.2%)	

**Table 3:** Multivariable logistic regression models to assess risk factors for having at least 1 HR-HPV genotype at screening and at the most recent HPV test prior to vaccination.

Characteristics	Category	Risk of Having ≥1 HR-HPV Genotype at HPV Screening		Risk of Having ≥1 HR-HPV Genotype at the Most Recent HPV Test Before Immunization	
		Adjusted Odds Ratio (95% CI)	*p*-Value	Adjusted Odds Ratio (95% CI)	*p*-Value
Age (years)	≥35 vs. <35	0.33 (0.18, 0.59)	<0.001	0.34 (0.18, 0.58)	<0.001
Smoking	Yes vs. No	1.29 (0.83, 1.73)	0.35	0.89 (0.66, 1.21)	0.46
CD4 nadir	per 100 cells/µL higher	1.09 (1.00, 1.20)	0.06	1.04 (0.97, 1.12)	0.27
CD4 count	per 100 cells/µL higher	0.98 (0.92, 1.05)	0.61	1.00 (0.94, 1.06)	0.95
HIV-RNA	detectable vs. undetectable	0.93 (0.51, 1.82)	0.82	1.05 (0.6, 1.98)	0.87
Previous STI	Yes vs. No	1.19 (0.75, 1.94)	0.48	1.31 (0.94, 1.84)	0.12
Concomitant STI	Yes vs. No	2.74 (1.59, 5.08)	<0.001	1.68 (1.09, 2.67)	0.023

## Data Availability

The data presented in this study are available on request from the corresponding author due to privacy restrictions.

## Supplementary Materials

The following supporting information can be downloaded at https://www.mdpi.com/article/10.3390/microorganisms12122571/s1. Figure S1: Percentage and number of MSM who tested positive for the various HPV serotypes at their first HPV test; Figure S2: Percentage and number of MSM who tested positive for the various HPV serotypes at their last HPV test before immunization; Table S1: Characteristics of MSM with HIV at HPV testing.
