# Supplementary material for: High Prevalence of High-Risk HPV Among People with and Without HIV: Insights into Risk Factors for Tailored Screening Approaches
Source: Microorganisms. 2024 Dec 13;12(12):2571. doi: 10.3390/microorganisms12122571 (PMC11677103; doi:10.3390/microorganisms12122571)
Supplement: Supplementary file 1 [file microorganisms-12-02571-s001.zip › microorganisms-3318711-supplementary.pdf]

Supplementary figure S1. *Percentage and number of MSM who tested positive for the various HPV serotypes at their first HPV test.*

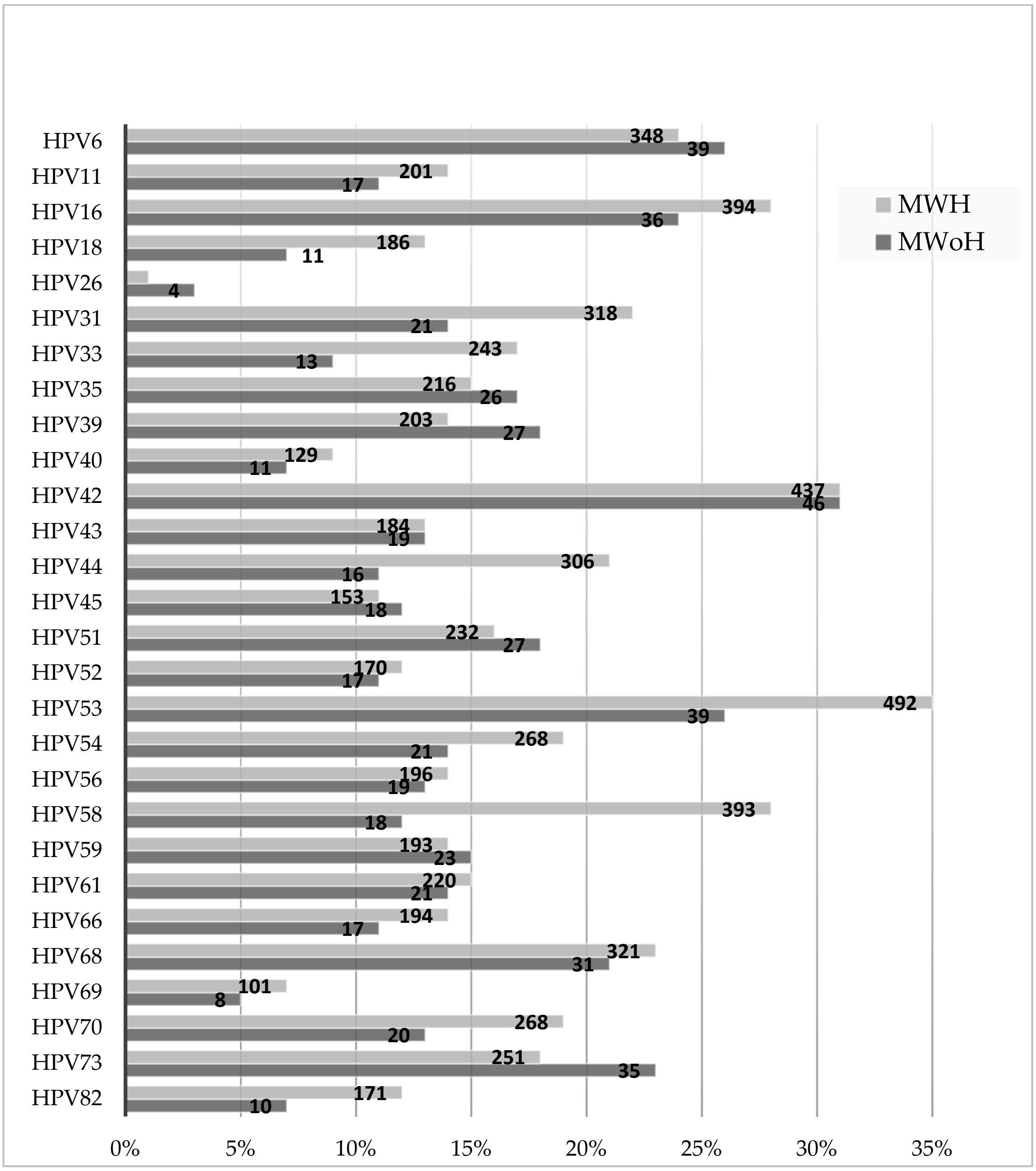

Supplementary figure S2. *Percentage and number of MSM who tested positive for the various HPV serotypes at their last HPV test before immunization.*

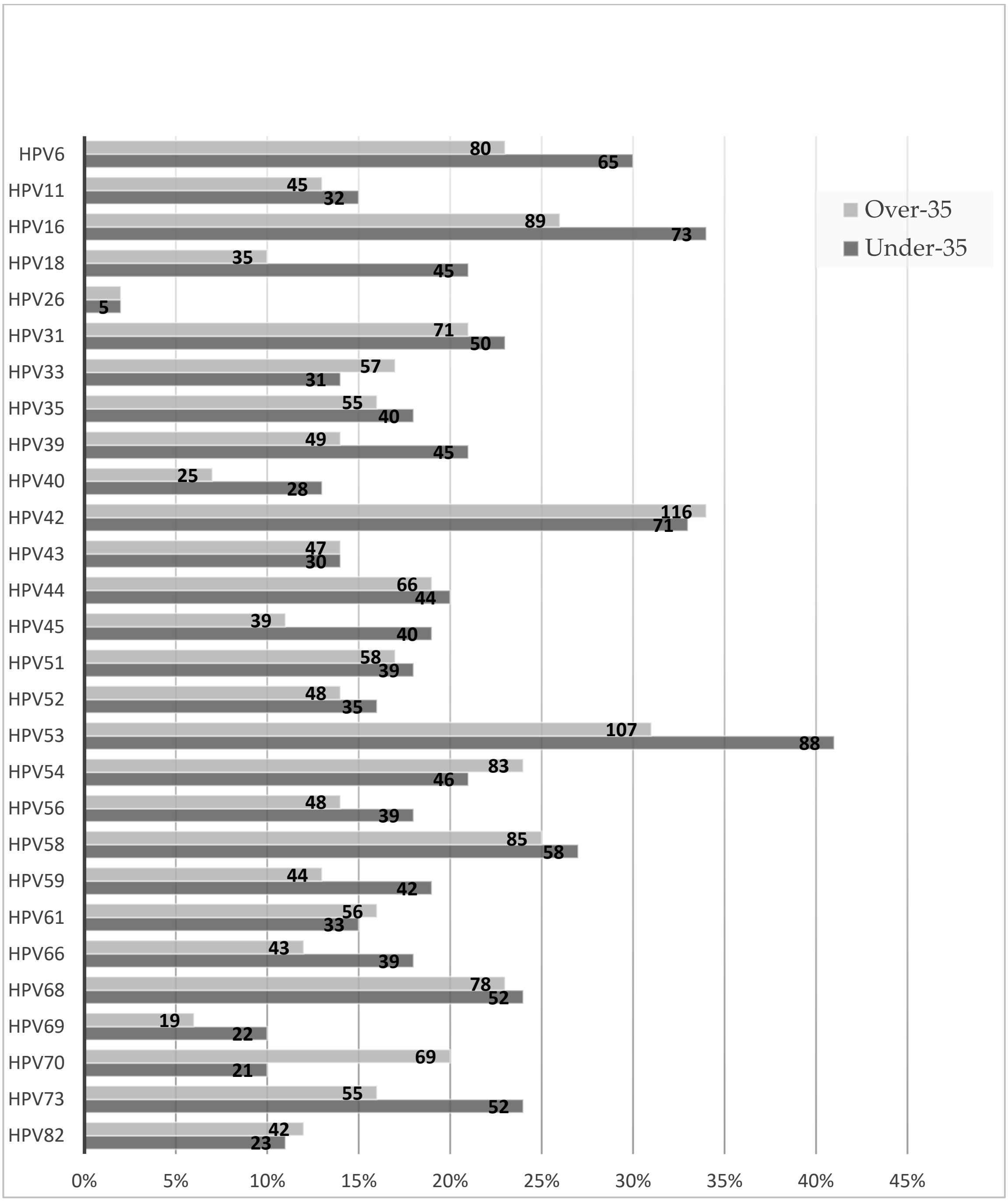

Supplementary Table S1. *Characteristics of MSM with HIV at HPV testing.*

	**MWH**<br>**N = 1427**
**Age**	44.5 [35.5;52.4]
**Age (2):**	
**<=45**	737 (51.6%)

**>45**	690 (48.4%)
**Years from HIV diagnosis**	8.51 [2.63;16.9]
**Naive:**	18 (1.26%)
**Years of ART**	6.54 [1.92;13.7]
**AIDS:**	154 (10.8%)
**HIV-RNA (copies/mL)**	0.90 [0.90;39.0]
**HIV-RNA below 40 cp/mL**	1158 (89.8%)
**Nadir CD4+ (cells/microL)**	355 [231;543]
**Nadir CD4+ (cells/microL) >200 cells/microL:**	1120 (80.3%)
**CD4+ (cells/microL)**	737 [554;940]
**CD4%**	31.4 [25.8;37.6]
**CD8+ (cells/microL)**	924 [690;1210]
**CD8%**	39.0 [32.8;46.8]
**CD4/CD8 ratio**	0.80 [0.56;1.10]
**ART regimen:**	
**On ART**	1393 (98.59%)
**Off-therapy**	2 (0.14%)
**Naive**	18 (1.27%)
